# The intracerebral hemorrhage acutely decreasing arterial pressure trial II (ICH ADAPT II) protocol

**DOI:** 10.1186/s12883-017-0884-4

**Published:** 2017-05-19

**Authors:** Laura Gioia, Ana Klahr, Mahesh Kate, Brian Buck, Dariush Dowlatshahi, Thomas Jeerakathil, Derek Emery, Kenneth Butcher

**Affiliations:** 1 Division of Neurology, University of Alberta, 7th Floor Clinical Sciences Building, 11350-83rd Avenue, Edmonton, AB T6G 2B7 Canada; 2 Division of Neurology, University of Ottawa, Ottawa, ON Canada; 3 Department of Diagnostic Imaging, University of Alberta, Edmonton, AB Canada

**Keywords:** Blood Pressure, Intracerebral Hemorrhage, DWI, MRI, Stroke, Clinical Trial

## Abstract

**Background:**

Aggressively lowering blood pressure (BP) in acute intracerebral hemorrhage (ICH) may improve outcome. Although there is no evidence that BP reduction changes cerebral blood flow, retrospective magnetic resonance imaging (MRI) studies have demonstrated sub-acute ischemic lesions in ICH patients. The primary aim of this study is to assess ischemic lesion development in patients randomized to two different BP treatment strategies. We hypothesize aggressive BP reduction is not associated with ischemic injury after ICH.

**Methods:**

The **I**ntra**c**erebral **H**emorrhage **A**cutely **D**ecreasing **B**lood **P**ressure **T**rial II (ICH ADAPT II) is a phase II multi-centre randomized open-label, blinded-endpoint trial. Acute ICH patients (*N* = 270) are randomized to a systolic blood pressure (SBP) target of <140 or <180 mmHg. Acute ICH patients within 6 h of onset and two SBP measurements ≥140 mmHg recorded >2 mins apart qualify. SBP is managed with a pre-defined treatment protocol. Patients undergo MRI at 48 h, Days 7 and 30, with clinical assessment at Day 30 and 90. The primary outcome is diffusion weighted imaging (DWI) lesion frequency at 48 h. Secondary outcomes include cumulative DWI lesion rate frequency within 30 days, absolute hematoma growth, prediction of DWI lesion incidence, 30-day mortality rates, day 90 functional outcome, and cognitive status.

**Discussion:**

This trial will assess the impact of hypertensive therapies on physiological markers of ischemic injury. The findings of this study will provide evidence for the link, or lack thereof, between BP reduction and ischemic injury in ICH patients.

**Trial registration:**

This study is registered with clinicaltrials.gov  (NCT02281838, first received October 29, 2014).

**Electronic supplementary material:**

The online version of this article (doi:10.1186/s12883-017-0884-4) contains supplementary material, which is available to authorized users.

## Background

Acute management of elevated blood pressure (BP) in acute intracerebral hemorrhage (ICH) remains an area of clinical equipoise. Phase III studies have failed to demonstrate marked improvements in clinical outcome when BP is lowered aggressively [1, 2]. The most recent trial demonstrated a trend to worse outcomes in patients in whom BP was lowered to 120–140 mmHg [2]. Although aggressive BP reduction has not been associated with lower cerebral perfusion [3], magnetic resonance imaging (MRI) studies have reported acute and subacute ischemic lesions in 14–41% of ICH patients [4–9]. These lesions have been associated with aggressive BP treatment as well as unfavorable clinical outcomes but these data are all retrospective [4, 5, 9]. Therefore, we designed a prospective randomized study to definitively determine whether acute BP reduction is associated with an elevated risk of ischemic injury, detected with diffusion-weighted imaging (DWI).

## Methods

### Study design

**Fig. 1:**
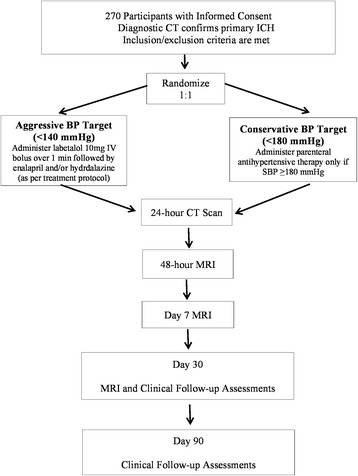
Study Schema

The **I**ntra**c**erebral **H**emorrhage **A**cutely **D**ecreasing **A**rterial **P**ressure **T**rial II (ICH ADAPT II) is a phase II multi-center prospective, randomized, open-label study with blinded-evaluation (PROBE). Eligible ICH patients are randomized 1:1 to systolic BP (SBP) targets of <140 or <180 mmHg (Fig. 1). This study is registered with clinicaltrials.gov (NCT02281838). The primary study aim is to assess DWI lesion frequency in patients randomized to aggressive versus conservative BP targets.

### Patient population

***Inclusion criteria***

- Patients ≥18 years with spontaneous ICH ≤6 h from onset
- Two SBP measurements ≥140 mmHg recorded >2 min apart
- Hematoma volume on CT <90 mL, as estimated using the ABC/2 method [10]
- Onset ≤6 h prior to randomization
- Glasgow Coma Scale (GCS) ≥5 prior to randomization

***Exclusion criteria***

- Definite contraindication to BP reduction (i.e. severe arterial stenosis, MoyaMoya disease)
- Definite indication for BP reduction (i.e. hypertensive encephalopathy or aortic dissection)
- Contraindication to MRI (i.e. cardiac pacemaker)
- Suspected secondary cause of ICH
- Ischemic stroke <90 days
- Planned hematoma resection
- Pre-morbid modified Rankin score (mRS) ≥3
- Life expectancy <6 months
- Early implementation of palliative care

### Randomization

All patients undergo standard screening assessments including BP, GCS, and National Institutes of Health Stroke Scale (NIHSS) scores prior to randomization (Table 1). A centralized web-based randomization process is used to assign BP treatment target.

**Table 1:** Summary of trial procedures

	Screening/Randomization	24 ± 3 h	48 ± 12 h	Day 7 ± 2	Day 30 ± 5	Day 90 ± 30
Eligibility Criteria	□					
Signed Informed Consent	□					
Past Medical History	□					
Vital Signs (BP, HR) Monitoring	□	□	□	□	□	□
Medications	□			□	□	□
CT scan	□	□				
MRI scan			□	□	□	
NIHSS	□	□	□	□	□	□
Glasgow Coma Scale	□	□	□	□	□	□
Modified Rankin Scale	□			□	□	□
Montreal Cognitive Assessment	□			□	□	□
Barthel Index, EuroQOL					□	□
AE/SAE Reporting		□	□	□	□	□
End of Study Report						□

### Interventions

#### BP Management

The assigned BP target is achieved and maintained using an intravenous antihypertensive drug bolus protocol (Table 2). Patients in the aggressive BP target (SBP <140 mmHg) group immediately receive a 10 mg intravenous (IV) bolus of labetalol, followed by repeated boluses designed to lower systolic BP to <140 mmHg within 60 min of randomization. The protocol utilizes IV enalapril and hydralazine boluses as well.

**Table 2:** Acute BP treatment protocols

A. <140 mmHg Target Group	
	Target SBP <140 mmHg within 30 min of randomization
Monitoring	● Record BP/HR q^a^5 min during active treatment; q15 min × 1 h, q30 min × 5 h and q1h × 18 h
Labetalol (IV)	● Labetalol test dose: 10 mg bolus over 1 min ● If SBP ≥140 mmHg and HR >55 BPM, repeat 10 mg bolus in 5 min. ● 10–20 mg IV push q5 min until SBP <140 mmHg or HR <55 BPM ● Maximum labetalol dose: 300 mg/24 h
And Enalapril (IV) (If available)	● Enalapril 1.25 mg bolus
And/or Hydralazine (IV)	If BP persistently >140 mmHg: ● Hydralazine test dose: 5 mg IV bolus over 1 min ● If SBP ≥140 mmHg, repeat 5 mg IV bolus in 5 min ● 10–20 mg IV bolus q5 min until SBP <140 mmHg ● Maximum hydralazine dose = 240 mg/24 h
Continuous IV Infusions (ICU admission)	If BP persistently >140 mmHg: ● Labetalol infusion 2–8 mg/min (maximum 300 mg/24 h) and/or hydralazine infusion 50–150 μg/min
Maintenance Therapy	
Maintain SBP <140 mmHg × 24 h minimum	
IV treatment prn^b^	If SBP >140 mmHg at any point: ● Labetalol (10–20 mg) / hydralazine (10–20 mg) boluses. Record BP/HR 5 and 15 min later ● Enalapril 1.25 mg q6 h if SBP >140 mmHg ● If SBP ≤135 mmHg or HR <55 BPM, hold maintenance dose
B. <180 mmHg Target Group	
	Protocol to be used only if SBP ≥180 mmHg
Monitoring	● as listed above
Labetalol (IV)	● Labetalol test dose: 10 mg bolus over 1 min ● If SBP ≥180 mmHg and HR >55 BPM, repeat 10 mg bolus in 5 min. ● 10–20 mg IV push q5 min until SBP <180 mmHg or HR <55 BPM ● Maximum labetalol dose: 300 mg/24 h
Hydralazine (IV)	If BP persistently >180 mmHg: ● Hydralazine test dose: 5 mg IV bolus over 1 min ● If SBP ≥180 mmHg, repeat 5 mg IV bolus in 5 min ● 10–20 mg IV bolus q5 min until SBP <180 mmHg ● Maximum hydralazine dose = 240 mg/24 h
Maintenance Therapy	
IV treatment prn	If SBP >180 mmHg at any point during 24 h: ● Labetalol (10–20 mg) / hydralazine (10–20 mg) boluses. Record BP/HR 5 and 15 min later

^a^q = every, ^b^prn = when necessary

Patients randomized to the conservative arm (SBP <180 mmHg group) are treated with antihypertensive agents only if SBP ≥180 mmHg. BP and heart rate (HR) are continuously monitored non-invasively for the first 24 h.

### Imaging Procedures

*Baseline:* Diagnostic non-contrast computed tomography (CT) scan*.* This consists of 5 mm slices, no gap (120 kvp, 300 mA per slice) through the entire brain (l8–20 slices with a 512 × 512 matrix).

*24-h:* Repeat CT scan at 24 ± 3 h to assess for hematoma expansion and peri-hematoma edema volume.

*48-h:* MRI at 48 ± 12 h, including a T1-weighted sagittal localizer, T2-weighted images, DWI (the primary endpoint), Fluid-attenuated Inverse Recovery (FLAIR), Susceptibility Weighted Imaging (SWI), and pulse Arterial Spin Labeling (ASL; optional sequence). DWI sequences are combined to form isotropic (trace) diffusion images and Apparent Diffusion Coefficient (ADC) maps are generated from these raw data. ADC maps are used to ensure all lesions represent true diffusion restriction and not T2 shine through effects. FLAIR sequences are also utilized to assess chronic small vessel ischemic changes. SWI sequences are used to assess the burden (number, total volume and topography) of cerebral microbleeds. Pulse ASL data is used to generate blood flow maps to determine if DWI lesions are correlated with hypoperfusion. All image analyses are completed centrally by raters blinded to BP treatment group allocation.

*Days 7 and 30:* MRI repeat at Days 7 ± 2 and 30 ± 5 to assess for new DWI lesion development and evolution of those previously identified.

### Clinical assessments

In-hospital clinical assessments (24 h, 48 h, day 7 and hospital discharge) of neurological deterioration are performed using GCS and NIHSS. Discharge mRS scores is obtained to assess disability and Montreal Cognitive Assessment (MoCA) to determine cognitive changes. This latter assessment is relevant as development of DWI lesions may impair cognition in ICH patients. All neurological, disability, and cognitive assessments are repeated at days 30 and 90.

### Primary outcome

The primary endpoint is DWI lesion frequency on the 48-h MRI, which is the time point DWI lesions have most commonly been observed after ICH [4–9]. This is also the time point most relevant to acute BP reduction.

**Secondary outcomes**

- Hematoma growth at 24 h
  - *Hypothesi*s: The mean hematoma growth will be smaller in the SBP <140 mmHg group.
- Cumulative DWI lesion rate frequency within 30 days
  - *Hypothesis:* The rate of DWI lesion development will be higher in the SBP <140 mmHg group.
- Day 30 mortality rates
  - *Hypothesis:* DWI lesion development will predict mortality, which should be independent of BP randomization.
- Day 90 mRS scores
  - *Hypothesis:* Median mRS will be positively correlated with number of DWI lesions.
- Day 90 MoCA
  - *Hypothesis:* MoCA scores will be lower in patients with DWI lesions.

### Data safety monitoring body (DSMB)

The DSMB (Additional file 1) reviews the proportion of patients with neurological deterioration within 48 h (defined as an increase in NIHSS ≥4 points), 90-day mortality and all serious adverse events (SAE) after 33% and 67% of patients are enrolled. The committee may modify or stop the trial at any point.

### Sample size estimates

The sample size is based on an observed DWI lesion frequency of 26% in the <180 mmHg target group [4–9]. The odds ratio for DWI lesion occurrence is 1.03 per decrease in mmHg of mean arterial pressure (MAP) between baseline and the MRI scan [5]. In ICH ADAPT I, MAP in the aggressive treatment group decreased by an average of 28 mmHg at the time of the primary endpoint assessment. Assuming a similar treatment effect in ICH ADAPT II, the predicted effect of SBP reduction <140 mmHg is an odds ratio of DWI lesion incidence of 1.84. The trial has been powered to detect a 0.84 (relative) increase in the frequency of DWI lesions in the <140 mmHg target group. The predicted absolute increase in the proportion of patients with DWI lesions is 22%.

*Hypotheses:*

- H_0_: The proportion of patients with DWI lesions in the < 140 mmHg treatment arm will be ≤ 0.48.
- H_A_: The proportion of patients with DWI lesions in the < 140 mmHg treatment arm will be > 0.48.
  The primary analysis will be a one-sided test of proportions at the alpha = 0.025 level. A sample size of 180 evaluable patients will be required to reject the null hypothesis with 80% power (alpha = 0.025). To account for withdrawal of consent and missing data related to early death and MRI contraindications, the sample size has been increased to 270 patients.

### Statistical analyses

The primary endpoint will be tested using Fisher’s Exact test. There will be no interim analyses for efficacy or futility.

## Discussion

Elevated BP has been associated with hematoma growth, mortality, and disability after ICH [11]. However, the most recent clinical trial data have revived concerns about the safety of very aggressive BP reduction [2]. It is unknown if this trend to worse outcome is related to cerebral ischemic changes, but it is one possible mechanism [12]. There are mixed data regarding the development of ischemic lesions after ICH and its association with BP. Retrospective studies have suggested that there is a negative, positive, and even no correlation between the two [5, 8, 13, 14].

The current trial utilizes a physiological endpoint that is more sensitive to ischemic injury to determine whether aggressive BP reduction in ICH patients exacerbates DWI lesion development, in individual patients. We have previously conducted a trial, ICH ADAPT I, in which ICH patients were randomized to aggressive (SBP <150 mmHg) versus conservative (SBP <180 mmHg) BP management strategies [3]. We did not find a difference in peri-hematoma cerebral blood flow (CBF) in these two groups. Most importantly, the magnitude of BP change was not related to regional CBF [3]. In contrast, several studies indicate that delayed cerebral ischemia, evident on MRI scan only, is common within the first week after ICH [4–9]. Although the majority of peri-hematoma DWI hyperintensities result from plasma derived edema,[15] true diffusion restriction representing bioenergetic compromise in this region and topographically remote areas are also present in some patients [4–9]. These DWI lesions tend to be small but are still a subject of concern as longitudinal studies indicate the probability of death/dependence at one year is increased between five and six-fold in these patients [13, 16].

Canadian Stroke Best Practices recommendations for hyperacute stroke care recognize the safety of aggressively reducing SBP [17]. Furthermore, current American Heart Association/American Stroke Association guidelines advise lowering SBP <140 mmHg given its potential to improve patient prognosis in ICH patients, but more aggressive treatment is not encouraged [18]. These recommendations are based primarily on the results of two phase III studies which have failed to demonstrate an increase in the proportion of patients with good clinical outcomes when BP is lowered aggressively [1, 2]. In the Intensive Blood Pressure Reduction in Acute Cerebral Hemorrhage Trial (INTERACT II), 2839 ICH patients were randomized within 6 h of symptom onset to a SBP target of <140 or <180 mmHg. The rate of death/disability was similar in the <140 mmHg target group (52%) and the <180 mmHg target group (55.3%; odds ratio with intensive treatment, 0.87; 95% CI, 0.75 to 1.01; *p* = 0.06) [1]. In the Antihypertensive Treatment of Acute Cerebral Hemorrhage II (ATACH II), patients were randomized earlier (4.5 rather than 6 h after symptom onset) to the aggressive and conservative BP strategies [2]. This trial recruited 1000 ICH patients, in which the primary outcome of death and disability (GCS >5) was observed in 38.7% vs. 37.7% in the aggressive vs. conservative treatment, respectively. The investigators concluded that aggressive antihypertensive therapy did not improve outcome.

At this point the optimal BP treatment strategy remains unknown. It has been hypothesized that an earlier intervention (e.g., in the prehospital setting) may be needed to improve patient prognosis, and small clinical trials have demonstrated the feasibility and safety of hyperacute BP management in stroke patients [19, 20]. However, it may be that in some patients aggressive BP reduction precipitate and/or exacerbate ischemic lesion development. This can only be addressed with a randomized controlled trial specifically addressing this endpoint.

In conclusion, ICH ADAPT II will provide evidence for the link, or lack thereof, between BP reduction and ischemic injury. This will add support for the safety of early systolic BP reduction to <140 mmHg, or alternatively a more nuanced approach to acute hypertension management in ICH patients.

## Additional files

Additional file 1: Data Safety Monitoring Body Members. (DOC 25 kb)

Additional file 2: Participant Information Sheet and Consent Form. (DOC 51 kb)

Additional file 3: Deferred Consent for Research Participation. (DOC 29 kb)

## Abbreviations

ADC: Apparent diffusion coefficient
ASL: Arterial spin labeling
ATACH II: Antihypertensive treatment of acute cerebral hemorrhage II
BP: Blood pressure
CBF: Cerebral blood flow
CT: Computed tomography
DSMB: Data safety monitoring body
DWI: Diffusion-weighted imaging
FLAIR: Fluid-attenuated inverse recovery
GCS: Glasgow coma scale
ICH ADAPT II: Intracerebral Hemorrhage Acutely Decreasing Blood Pressure Trial II
ICH: Intracerebral hemorrhage
INTERACT II: Intensive blood pressure reduction in acute cerebral hemorrhage II
MAP: Mean arterial pressure
MoCA: Montreal cognitive assessment
MRI: Magnetic resonance imaging
mRS: Modified Rankin scale
NIHSS: National Institutes of Health Stroke Scale
PROBE: Prospective, randomized, open-label study with blinded-evaluation
SAE: Serious adverse events
SBP: Systolic blood pressure
SWI: Susceptibility Weighted Imaging
