# Supplementary material for: The intracerebral hemorrhage acutely decreasing arterial pressure trial II (ICH ADAPT II) protocol
Source: BMC Neurol. 2017 May 19;17:100. doi: 10.1186/s12883-017-0884-4 (PMC5437568; doi:10.1186/s12883-017-0884-4)
Supplement: Supplementary file 1 — Data Safety Monitoring Body Members. (DOC 25 kb) [file 12883_2017_884_MOESM1_ESM.doc]

Additional file 1

Data Safety Monitoring Body Members

Dr. Georgios Tsivgoulis (MD, PhD, DSMB Chair) – Department of Neurology, University of Athens, Athens, Greece.

Dr. Vijay K. Sharma (MD) -  Yong Loo Lin School of Medicine, National University of Singapore, Singapore.

Dr. Christos Krogias (MD) - Department of Neurology, St. Josef-Hospital, Ruhr University, Bochum, Germany.
