# Supplementary material for: The intracerebral hemorrhage acutely decreasing arterial pressure trial II (ICH ADAPT II) protocol
Source: BMC Neurol. 2017 May 19;17:100. doi: 10.1186/s12883-017-0884-4 (PMC5437568; doi:10.1186/s12883-017-0884-4)
Supplement: Supplementary file 2 — Participant Information Sheet and Consent Form. (DOC 51 kb) [file 12883_2017_884_MOESM2_ESM.doc]

Additional file 2

**PARTICIPANT INFORMATION SHEET**

**Study Title**: **The Intracerebral Hemorrhage Acutely Decreasing Arterial Pressure Trial II (ICH ADAPT II)**

**Principal Investigator**: Dr. Ken Butcher (780-248-1927)

**Co-investigators**: Dr. Thomas Jeerakathil, Dr. Ashfaq Shuaib, Dr. Max Findlay, Dr. Maher Saqqur, Dr. Derek Emery, Dr. Brian Buck, Dr. Hayrapet Kalashyan, Dr. Alejandro Manosalva, Dr. Andrew Wassef, Dr. Bashir Brebesh

**Research Coordinator:** Leka Sivakumar (office: 780-248-1118)

______________________________________________________________________________

If you are giving consent to participate on behalf of the patient, the word “you” may at times be interpreted as “your relative” and/or “substitute decision maker”.

**Why am I being asked to take part in this research study?**

You have had a type of stroke, known as intracerebral hemorrhage (ICH). This is a name for bleeding into the brain. You are asked to take part in a research study because you have had a intracerebral hemorrhage within the last 6 hours. You also have high blood pressure. Doctors are unsure how best to treat high blood pressure right after stroke. Some doctors think lower blood pressures are better, while others think that high blood pressure should not be treated too early. The purpose of this study is to determine if lowering blood pressure early is safe.

Treatment for this study is required to start within 6 hours of the onset of a stroke, so you may only have a short time to think about whether or not to take part. If you need an interpreter, this can be arranged within the available time frame.

**What will I be asked to do?**

You have already had a CT scan (special x-ray of your head) that shows the bleed in your brain. If you agree to take part in the study you will be assigned to one of two groups. The assignment will be random (like the flip of a coin). Blood pressure is measured in mm of mercury (mmHg). Depending on the group you are in, your blood pressure may be kept lower than 140 mmHg or lower than 180 mmHg. You and your doctor will know which of the treatment groups you are assigned to. Your blood pressure will be lowered with medications given to you intravenously (IV). You will have another CT scan tomorrow to see if bleeding in your brain has increased. You will have this scan even if you do not participate in the trial. After that, your blood pressure will be treated as your doctor normally would. This usually means giving you pills to take for the rest of your life. You will also have up to three MRI scans of your brain. The first will be done 2 days after the stroke, the second one a week from the stroke, and the third in a month. At each of these visits we will gather information about you. We will ask you questions and use standard tests to assess your well-being. We will draw some blood from you. Tests will be done on your blood that may help identify additional factors associated with the type of stroke you have had. Dr. Ken Butcher or his designate will see you about 3 months after your stroke. There will be no MRI but the same questions and tests will be used to assess you.

**What are the risks to me?**

All medical procedures involve some risk of injury. There may be risks associated with this study that are unknown and cannot be predicted. All precautions will be taken to avoid this. The possible risks of participating in this study include:

***Medication:*** All medications used in this study are approved for use in Canada. No experimental drugs will be used. Blood pressure lowering drugs may cause side effects. Less than 10% of individuals will experience these side effects. These usually mild and may resolve immediately by reducing or stopping the drug. These side effects may include dizziness, headache, vomiting and other drug-specific side effects. As with any medication, an allergic reaction to blood pressure lowering drugs is possible, but also quite rare. If you have any questions or concerns about your medications please talk to your doctor or nurse.

***Intravenous line or blood tests:*** At the site of needle insertion there may be temporary bruising, discomfort, or irritation. There is also a small risk of infection whenever a needle breaks the skin. Even if you were not in the study, you will have an intravenous line and blood tests done.

***CT Scans:*** This is a special x-ray used to generate pictures of the brain. You just had one, which showed your stroke. The scanning process is totally painless and has no significant harmful effects. The scan takes a few minutes. During the scan, you will be asked to lie on a special table, which moves into the machine. You need to keep still during the examination. The person operating the machine will talk to you through an intercom telling you what is happening, and when they are starting each new set of scans. A CT involves exposure to a small dose of x-ray radiation. As part of everyday living, everyone is exposed to naturally occurring background radiation. Background radiation is increased when we take long flights in jet aircraft. The radiation dose from a CT scan of the head is about the same as you would receive living in Edmonton for about 3 years. At this dose level, no harmful effects of radiation have been demonstrated and the risk is negligible. You have already had one CT scan of the head. You will have one more scan tomorrow. These are both considered standard of care, meaning your doctor would order them even if you were not in the study.

***MRI Scans:*** An MRI (Magnetic Resonance Imaging) machine uses magnets to generate pictures of the brain. The scanning process is totally painless and has no significant harmful effects. The scan takes about 20 minutes. During the scan, you will be asked to lie on a special table, which moves into the machine. You need to keep still during the examination. The space inside the machine is small, which can make some people uneasy. The person operating the machine will talk to you through an intercom telling you what is happening, and when they are starting each new set of scans.

With the first two scans you may also have an injection of contrast ‘dye’ used to measure blood flow in the brain. Possible side-effects of this dye include a flushing sensation, a salty or metallic taste in the mouth, brief headache, or nausea/vomiting. These effects are usually mild and only last for a few moments. An allergic reaction to the dye is possible. This is usually mild and easily treated. About 2 in 1000 people have a bad reaction to the dye. A very serious reaction to the dye has been reported in some patients with kidney problems. You will have had tests for kidney failure before being asked to participate in this study. If you have even mild kidney problems, you will not receive any dye, for this reason. Sometimes the dye leaks into the arm and this can be painful. This is rare and we avoid it by using a big vein in the arm.

Your participation in this study will be stopped should any harmful effects appear or if the doctor feels it is not in your best interest to continue. Your doctor may at any time provide you with any other treatment he/she considers necessary.

**What are the benefits to me?**

This study will further medical knowledge and may improve treatment of stroke in the future, but a direct benefit to you cannot be guaranteed. At this point, the best way to treat blood pressure is unknown.

**What happens if I am injured because of this research?**

If you become ill or injured as a result of participating in this study, necessary medical treatment will be available at no additional cost to you. By signing this consent form you are not releasing the investigators or institution from their legal and professional responsibilities.

**Do I have to take part in this study?**

You do not have to be in this study to receive care. You are free to withdraw from the research study at any time, and your continuing medical care will not be affected in any way. If the study is not undertaken or if it is discontinued at any time, the quality of your medical care will not be affected. If any knowledge gained from this or any other study becomes available which could influence your decision to continue in the study, you will be promptly informed.

**Will I be paid for participating or will I receive any funds?**

There will be no cost to you for participating, and you will not be paid for participating. Your parking costs for return visits will be reimbursed.

**Will my information be kept private?**

During the study we will be collecting health data about you. We will do everything we can to make sure that this data is kept private. Sometimes, by law, we may have to release your information with your name so we cannot guarantee absolute privacy. However, we will make every legal effort to make sure that your health information is kept private.

No data relating to this study that includes your name will be released outside of the study doctor’s office or published by the researchers. The CT/MRI images (pictures) collected through this research will be stored confidentially in a local computerized database that is protected by a “firewall”. A database allows researchers to evaluate the information that was collected at a later time. Also further examination of the information and secondary publications may be made to help increase the knowledge of stroke, but none will identify you by name.

The study doctor/study staff may need to look at your personal health records held at the study doctor’s office, and/or kept by other health care providers that you may have seen in the past (i.e. your family doctor). Any personal health information that we get from these records will be only what is needed for the study.

During research studies it is important that the data we get is accurate. For this reason your health data, including your name, may be looked at by people from the University of Alberta auditors or members of the Research Ethics Board, Health Canada, and/or other foreign regulatory agency.

After the study is done, we will still need to securely store your health data that was collected as part of the study. In Canada, the law says we have to keep the data stored for 25 years after the end of the study; after which the all the study data will be destroyed.

By signing the consent form you are giving permission for the study doctor/staff to collect, use and disclose information about you from your personal health records as described above.

**What if I have questions?**

If you have questions about the research now or later, please contact Dr. Ken Butcher (780-248-1927) or the study coordinator (office: 780-248-1118)

If you have concerns about your rights as a study participant, you may contact the Research Ethics Office at 780-492-2615. This office has is independent of the study investigators.

**PARTICIPANT CONSENT FORM**

**Study Title: The Intracerebral Hemorrhage Acutely Decreasing Arterial Pressure Trial II (ICH ADAPT II)**

**Principal Investigator**: Dr. Ken Butcher (780-248-1927)

**Co-Investigators**: Dr. Thomas Jeerakathil, Dr. Ashfaq Shuaib, Dr. Max Findlay, Dr. Maher Saqqur. Dr. Derek Emery, Dr. Brian Buck, Dr. Hayrapet Kalashyan, Dr. Alejandro Manosalva, Dr. Andrew Wassef, Dr. Bashir Brebesh

Yes No

Do you understand that you have been asked to be in a research study? 🞎 🞎

Have you read and received a copy of the attached Information Sheet? 🞎 🞎

Do you understand the benefits and risks involved in taking part in this research 🞎 🞎

study?

Have you had an opportunity to ask questions and discuss this study? 🞎 🞎

Do you understand that you are free to withdraw from the study at any time, 🞎 🞎

without having to give a reason and without affecting your future medical care?

Has the issue of confidentiality been explained to you? 🞎 🞎

Do you understand who will have access to your records, including 🞎 🞎

personally identifiable health information?

Do you want the investigator(s) to inform your family doctor that you are 🞎 🞎

participating in this research study? If so, give his/her name ________________________

Who explained this study to you? __________________________________________________

I agree to take part in this study: YES 🞎 NO 🞎

Printed Name Signature of Participant Date

Substitute decision maker (printed) Signature Relationship Date

Signature of Witness (to SDM): **____________________** Date_______________________

I believe that the person signing this form understands what is involved in the study and

voluntarily agrees to participate.

Signature of Person Obtaining Consent:_____________________ Date: ______________________

THE INFORMATION SHEET MUST BE ATTACHED TO THIS CONSENT FORM AND A COPY GIVEN TO THE RESEARCH PARTICIPANT

**REGAINED CAPACITY CONSENT FORM**

**Study Title: The Intracerebral Hemorrhage Acutely Decreasing Arterial Pressure Trial II (ICH ADAPT II)**

**Principal Investigator**: Dr. Ken Butcher (780-248-1927)

**Co-Investigators**: Dr. Thomas Jeerakathil, Dr. Ashfaq Shuaib, Dr. Max Findlay, Dr. Maher Saqqur. Dr. Derek Emery, Dr. Brian Buck, Dr. Hayrapet Kalashyan, Dr. Alejandro Manosalva, Dr. Andrew Wassef, Dr. Bashir Brebesh

Because your illness or injury made it impossible for you to participate fully in the informed consent process, the consent was obtained from your surrogate on your behalf. Your surrogate believed you would have wished to participate in this research if you had been able to express your own opinion at the beginning of the research project.

Informed consent is essential throughout a research project. This means in your situation, you are now being given the opportunity to agree or disagree with the decision made by your surrogate for you to participate. Any information that was obtained before when the researchers were acting on your surrogate’s consent for your involvement will remain part of the study information, but it is now your choice whether to continue.

Please indicate one of the following options with a check mark:

I wish to remain in this study. I have read and reviewed the

information and consent forms originally signed on my behalf 🞎

and my questions have been answered

I wish to withdraw from this study. 🞎

Signature of Research Subject _______________________________________________________

(Printed Name) ________________________________________ Date______________________

Signature of Investigator or Designee: ___________________________ Date: ________________

THE INFORMATION SHEET MUST BE ATTACHED TO THIS CONSENT FORM AND A COPY GIVEN TO THE RESEARCH PARTICIPANT
