# Supplementary material for: The intracerebral hemorrhage acutely decreasing arterial pressure trial II (ICH ADAPT II) protocol
Source: BMC Neurol. 2017 May 19;17:100. doi: 10.1186/s12883-017-0884-4 (PMC5437568; doi:10.1186/s12883-017-0884-4)
Supplement: Supplementary file 3 — Deferred Consent for Research Participation. (DOC 29 kb) [file 12883_2017_884_MOESM3_ESM.doc]

Additional file 3

**Deferred Consent for Research Participation**

**Title of Project:** **The Intracerebral Hemorrhage Acutely Decreasing Arterial Pressure Trial II (ICH ADAPT II)**

**Principal Investigator:** Dr. Ken Butcher (780-248-1927)

**Co-Investigators:** Dr. Thomas Jeerakathil, Dr. Ashfaq Shuaib, Dr. Max Findlay, Dr. Maher Saqqur. Dr. Derek Emery, Dr. Brian Buck, Dr. Hayrapet Kalashyan, Dr. Alejandro Manosalva, Dr. Andrew Wassef, Dr. Bashir Brebesh

**Research Coordinator: Leka Sivakumar** (office: 780-248-1118)

The patient named below is being enrolled in this research study by deferred consent.

When a previously incapacitated participant regains capacity, or when a substitute decision maker is found, consent shall be sought promptly for continuation in the study, and for subsequent examinations or tests related to the research study. Seeking consent prior to study participation is always preferable.

If no substitute decision maker/family member is available on site or they refuse to give consent, any data or samples collected for study purposes will be destroyed.

**Patient’s Name:** _____________________________________________________

**Date/time assessed for enrolment:** _____/______/______ (dd/mm/yyyy) at ____ : ______ (24hr time)

**Reason(s) deferred consent process is used (check all that apply):**

____ The patient is unconscious or lacks capacity to understand the risks, methods and purposes of the research study.

____ No next of kin/substitute decision maker is available to provide consent, or attempts to contact them have been unsuccessful despite diligent and documented efforts.

____ A substitute decision maker ___________________________________ (name and relationship) has been contacted by telephone, and the purpose, methods and risks of participation in this study have been explained to the third party. While the substitute decision maker has given verbal consent for participation, written consent must be still be obtained.

____ No relevant prior directive by the patient is known to exist.

____ Other: ___________________________________________________________________

______________________________________________ ___________________________

**Signature of investigator/designee Date and Time**
